# Models Estimating the Effects of Changes in the Concentration of Specific Climatic Pollutants and Temperature on the Prevalence of Acute Ophthalmic Inflammatory Conditions: A Retrospective Cross‐Sectional Observational Study

**DOI:** 10.1155/joph/7498511

**Published:** 2026-08-23

**Authors:** Monika Sarnat-Kucharczyk, Dorota Wyględowska-Promieńska, Sudi Patel

**Affiliations:** ^1^ Department of Ophthalmology, Faculty of Medical Sciences in Katowice, Medical University of Silesia, Katowice, Poland, sum.edu.pl; ^2^ Kornel Gibinski University Clinical Centre, Katowice, Poland; ^3^ Department of Cataract and Refractive Surgery, University Eye Clinic Svjetlost, Zagreb 10000, Croatia

**Keywords:** acute ophthalmic inflammatory conditions, air pollution, temperature

## Abstract

**Background:**

Air pollution is a major environmental health risk linked to systemic and ocular diseases, including conjunctivitis, keratitis, and dry eye disease. Pollutants such as particulate matter, nitrogen oxides, and diesel exhaust particles have been associated with an increased prevalence of acute ophthalmic inflammatory conditions (AOICs). Identifying key pollutants may help reduce the prevalence of AOICs.

**Aim:**

The aim of this study was to investigate the relationship between atmospheric pollution and AOICs by identifying pollutants with the most consistent impact on AOIC prevalence, developing predictive models to estimate their composite effect, and assessing model accuracy.

**Methods:**

This retrospective study analyzed cases of AOICs from the Eye Emergency Department in Katowice (2011–2019). Diagnoses were classified into subgroups using ICD‐10 codes and ophthalmologic evaluation. Annual prevalence was correlated with average pollutant levels and temperature via linear and multiple regression. Significant associations informed predictive models, assessed for accuracy using limits of agreement (±1.96 × SD), with *p* < 0.05 as the significance threshold.

**Results:**

Between 2011 and 2019, over 60,000 cases of AOICs were identified among nearly 95,000 emergency visits. The most frequent diagnoses included conjunctivitis (46.8%), keratitis (26.4%), and blepharitis (14.1%). Multiple regression analyses revealed that the prevalence of several AOIC subtypes was significantly associated with mean annual concentrations of specific pollutants and/or temperature.

**Conclusion:**

Formaldehyde, CO, SO_2_, PM_2_._5_, and PM_10_ were significantly linked to inflammatory ocular conditions, with stronger associations after excluding noninflammatory cases and conjunctivitis. These findings highlight the role of environmental monitoring in predicting or reducing serious eye inflammation, supporting further large‐scale research.

## 1. Introduction

Air pollution poses a serious threat to human health, and studies have confirmed the negative impact of pollution on various systemic and ocular diseases [1–3]. Despite its various types, air pollution is increasingly recognized as a significant environmental factor that is gaining attention among researchers due to its widespread health consequences affecting multiple organs [4–6]. Air pollution also poses a particularly severe threat to children. According to WHO data, approximately 1 in 10 deaths among children under the age of five is attributed to exposure to air pollution [7]. Air pollutants encompass particulate matter (PM), diesel exhaust particles (DEPs), and various gases, including hydrocarbons, nitrogen oxides (NOx—primarily nitric oxide [NO] and nitrogen dioxide [NO_2_]), sulfur dioxide (SO_2_), and carbon monoxide (CO). PM is categorized based on its diameter: PM_10_ ranges from 2 to 10 μm, while PM_2.5_ measures between 0.5 and 2.5 μm. PM_10_ primarily consists of dust, pollen, and mold, whereas PM_2.5_ includes combustion byproducts, organic compounds, and metallic particles. DEPs belong to the category of ultrafine PM, with a diameter of less than 0.1 μm.

Harmful airborne particles have been linked to conditions such as dry eye disease (DED), glaucoma, cataracts, conjunctivitis, age‐related macular degeneration, and corneal inflammation [8–10].

A recent paper [11] reported on the associations between local atmospheric levels of a range of pollutants, annual temperatures, and the prevalence of acute ophthalmic inflammatory conditions (AOICs). Significant, direct and positive correlations were detected between a host of pollutants and the prevalence of individual AOICs. This suggests that it may be possible to curtail the prevalence of AOICs by reducing the levels of airborne pollutants. A practical solution would be to identify the specific pollutant or combination of pollutants that have the greatest impact on individual AOICs.

The study was conducted in Katowice, the largest academic and healthcare center in the Silesian Voivodeship, Southern Poland [12]. The city is located within a densely populated and highly urbanized region with a long history of coal mining, metallurgy, and energy production [13]. Consequently, air pollution has been a major environmental and public health concern in this area for decades. Silesian Voivodeship has the highest population density in Poland (∼379 inhabitants/km^2^) and is the most industrialized and urbanized region of the country. Although pollutant concentrations have decreased over time, exceedances of PM limits continue to be reported in Katowice and surrounding municipalities.

Previous studies have reported associations between air pollution and ocular diseases in different geographical settings. In Japan [14], exposure to airborne PM was associated with increased rates of allergic conjunctivitis and ocular surface irritation. In Brazil [15], chronic exposure to traffic‐related air pollution was linked to tear film instability and ocular surface changes. Similarly, systematic reviews have demonstrated associations between air pollution and DED, conjunctivitis, and blepharitis [16, 17]. However, the strength and direction of these associations vary between studies, likely reflecting differences in climate, pollutant composition, industrial activity, population characteristics, and study design.

While previous studies have primarily examined individual ocular disorders or selected ocular surface outcomes, the present study addresseses this gap by evaluating the relationship between environmental factors and the overall prevalence of AOICs and by exploring whether these associations can be incorporated into predictive models.

Furthermore, evidence from heavily industrialized regions of Central and Eastern Europe is limited. Therefore, data obtained from the Silesian region may provide valuable additional insight into any relationship between environmental pollution and ocular inflammation.

We hypothesized that variations in environmental conditions may influence the prevalence of AOICs and that these relationships could be incorporated into predictive models.

The aim of this study was to:

i. Identify the specific atmospheric pollutants that have the most consistent impact on AOICs;
ii. Determine whether models could be formulated to predict the composite effect of specific atmospheric pollutants on the prevalence of AOICs;
iii. Determine the likely accuracy and margin of error associated with any predictive models.

## 2. Methods

### 2.1. Study Design and Setting

A retrospective cross‐sectional observational study of AOICs was conducted on patients who presented to the Eye Emergency Department at the Professor K. Gibinski University Clinical Center, Medical University of Silesia, Katowice, Poland, between 01/01/2011 and 31/12/2019. The hospital is a tertiary referral academic center serving the Silesian Voivodeship in southern Poland. This is the most densely populated region in Poland, with approximately 4.3 million inhabitants, and has a long history of heavy industrial activity, mining, and energy production. Katowice is located within the Upper Silesian metropolitan area, one of the largest metropolitan areas in Central Europe. The region has historically been affected by elevated levels of air pollution, making it a relevant setting for investigating potential associations between environmental factors and ophthalmic disease.

The period 2020–23 was excluded because of the disruption arising from the COVID–19 pandemic that resulted in a conspicuous decline in emergency department attendance during this period.

### 2.2. Participants

Medical records were reviewed annually. The analysis proceeded in three stages: (1) the total number of patients presenting each year was documented, (2) cases diagnosed with AOICs were identified, and (3) these cases were categorized into the following diagnostic subgroups: blepharitis, orbital inflammation, lacrimal system inflammation, conjunctivitis, keratitis, uveitis with retinitis, endophthalmitis, optic neuritis, and scleritis. Inclusion criteria were based on diagnoses coded according to the International Classification of Diseases, 10th Revision (ICD‐10), as summarized in Table 1, and on detailed clinical assessments performed by ophthalmologists at the time of presentation. These data were derived from a previous study [11].

**TABLE 1:** International Classification of Disease (ICD) codes and descriptions during the study period [7].

Condition	ICD‐10 code
Blepharitis	H01.0, H01.001, H01.002, H01.003, H01.004, H01.005, H01.006, H01.009, H01.011, H01.012, H01.013, H01.014, H01.015, H01.016, H01.019, H01.021, H01.022, H01.023, H01.024, H01.025, H01.026, H01.029
Lacrimal system inflammation	H04.0, H04.001, H04.002, H04.003, H04.009, H04.011, H04.012, H04.013, H04.019, H04.101, H04.102, H04.103, H04.109, H04.301, H04.302, H04.303, H04.309, H04.401, H04.402, H04.403, H04.409, H04.411, H04.412, H04.413, H04.419, H04.201, H04.202, H04.203, H04.209
Orbital inflammation	H05.0, H05.001, H05.002, H05.003, H05.009, H05.101, H05.102, H05.103, H05.109
Conjunctivitis	H10.0, H10.011, H10.012, H10.013, H10.019, H10.021, H10.022, H10.023, H10.029, H10.101, H10.102, H10.103, H10.109, H10.201, H10.202, H10.203, H10.209, H10.401, H10.402, H10.403, H10.409, H10.801, H10.802, H10.803, H10.809, H10.901, H10.902, H10.903, H10.909, H10.301, H10.302, H10.303, H10.309, H10.5
Scleritis	H15.0, H15.001, H15.002, H15.003, H15.009, H15.101, H15.102, H15.103, H15.109, H15.201, H15.202, H15.203, H15.209
Keratitis	H16.0, H16.001, H16.002, H16.003, H16.004, H16.011, H16.012, H16.013, H16.014, H16.021, H16.022, H16.023, H16.024, H16.031, H16.032, H16.033, H16.034, H16.041, H16.042, H16.043, H16.044, H16.051, H16.052, H16.053, H16.054, H16.101, H16.102, H16.103, H16.104, H16.201, H16.202, H16.203, H16.204, H16.211, H16.212, H16.213, H16.214, H16.221, H16.222, H16.223, H16.224, H16.231, H16.232, H16.233, H16.234, H16.301, H16.302, H16.303, H16.304, H16.311, H16.312, H16.313, H16.314, H16.8, H16.9
Uveitis with retinitis	H30.9, H30.00, H30.01, H30.02, H30.03, H30.10, H30.11, H30.12, H30.13, H30.20, H30.21, H30.22, H30.23, H30.90, H30.91, H30.92, H30.93
Endophthalmitis	H44.0, H44.021, H44.022, H44.023, H44.029, H44.031, H44.032, H44.033, H44.039, H44.041, H44.042, H44.043, H44.049, H44.091, H44.092, H44.093, H44.099
Optic neuritis	H46.0, H46.00, H46.01, H46.02, H46.03, H46.1, H46.11, H46.12, H46.13

### 2.3. Variables and Data Sources

#### 2.3.1. Temperature

Annual average temperature data were obtained from official online sources, including national‐level statistics for Poland provided by the International Energy Agency (https://www.iea.org/articles/poland-climate-resilience-policy-indicator) and regional data for Silesia from Weather and Climate (https://weatherandclimate.com/poland/silesia) [18, 19].

#### 2.3.2. Pollution

Data on air pollutant levels for the Silesian region were obtained from the official reports published online by the Chief Inspectorate of Environmental Protection (Główny Inspektorat Ochrony Środowiska–GIOŚ) (https://www.gov.pl/web/gios-en), covering the period from 2010 to 2019 [20]. Air pollution data were analyzed at the regional level. Annual mean concentrations reported by GIOŚ were used as measures of population‐level environmental exposure in the Silesian Voivodeship. These annual values were matched with the corresponding annual prevalence of AOICs recorded at the Eye Emergency Department. The objective was to identify composite associations between environmental factors and the annual prevalence of AOICs rather than to assess individual patient response to exposure.

The pollutants reported by GIOŚ and considered for inclusion in this study are summarized in Table 2.

**TABLE 2:** Pollutants considered and listed by chief inspectorate of environmental protection [10].

Pollutant	Abbreviation
Sulfur dioxide (μg/m^3^)	SO_2_
Nitric oxide (μg/m^3^)	NO
Nitrogen dioxide (μg/m^3^)	NO_2_
Nitrogen oxides (μg/m^3^)	NO_x_
Carbon monoxide (mg/m^3^)	CO
Ozone (μg/m^3^)	O_3_
Benzene (μg/m^3^)	C_6_H_6_
Particulate matter in suspended dust with a diameter ≤ 10 microns (μg/m^3^)	PM_10_
Particulate matter in suspended dust with a diameter ≤ 2.5 microns (μg/m^3^)	PM_2.5_
Lead in suspended dust particles with a diameter ≤ 10 microns (μg/m^3^)	Pb(PM_10_)
Arsenic in suspended dust particles with a diameter ≤ 10 microns (ng/m^3^)	As(PM_10_)
Cadmium in suspended dust particles with a diameter ≤ 10 microns (ng/m^3^)	Cd(PM_10_)
Nickel in suspended dust particles with a diameter ≤ 10 microns (ng/m^3^)	Ni(PM_10_)
Benzo(a)pyrene in suspended dust particles with a diameter ≤ 10 microns (ng/m^3^)	BaP(PM_10_)
Polycyclic aromatic hydrocarbons (PAHs) in suspended dust particles with a diameter ≤ 10 microns (ng/m^3^)	PAHs(PM_10_)
Ions in PM_2.5_ in suspended dust particles with a diameter ≤ 2.5 microns (μg/m^3^)	Ions(PM_2.5_)
Total gaseous mercury (ng/m^3^)	Hg(TGM)
Formaldehyde (μg/m^3^)	Not abbreviated

### 2.4. Efforts to Address Potential Sources of Bias

All patients presenting with AOICs that attended the emergency department between 2011 and 2019 were included to minimize selection bias. Diagnoses were consistently classified using ICD‐10 codes and confirmed by ophthalmologic evaluation to reduce misclassification. Annual average pollutant concentrations were used to limit the effect of short‐term variability. Potential confounding by seasonal viral infections or allergies could not be entirely excluded, but the large sample size and standardized diagnostic criteria helped to mitigate this risk.

### 2.5. Study Size

All available cases of AOICs presenting to the Eye Emergency Department in Katowice between 2011 and 2019 were included. No formal sample size calculation was performed, as the study included all eligible cases during the study period. The large number of cases (> 60,000) ensured adequate power for regression analyses.

### 2.6. Quantitative Variables

Pollutant concentrations (formaldehyde, CO, SO_2_, PM_2_._5_, and PM_10_) and mean annual temperature were analyzed as continuous variables using annual average values obtained from environmental monitoring stations. The outcome was the annual prevalence of AOICs, calculated as the proportion of cases among all emergency visits for each year. No categorical groupings were applied, as continuous modeling allowed a more precise assessment of linear associations.

### 2.7. Data and Statistical Analysis

Data were organized in Excel spreadsheets (Microsoft Corp., Redmond, WA, USA). Data sets for the prevalence of all AOICs, and each AOIC, among the total number attending the Emergency Department each year, was analyzed using linear regression analysis to determine the significance of any correlation between the following:

i. prevalence of all AOICs and of each AOIC, in relation to the mean annual concentration of each pollutant and mean annual temperature.
ii. prevalence of each AOIC after excluding (a) all noninflammatory conditions and (b) all noninflammatory conditions and conjunctivitis, in relation to the mean annual concentration of each pollutant and mean annual temperature.

For each AOIC outcome, simple linear regression analyses were first performed to assess associations with individual pollutants and mean annual temperature. Variables showing statistically significant associations (*p* < 0.05) were subsequently entered into multiple linear regression models. Separate models were developed for each AOIC subtype. Model performance was evaluated using the coefficient of determination (*r* ^2^), and statistical significance was set at *p* < 0.05.

If a significant correlation was detected between the prevalence of an AOIC and the concentrations of pollutants and mean annual temperature, the resulting regression equation was used to estimate the limits of agreement (LoA) between estimated and observed prevalence values over the period 2011 to 2019.

Nonparametric tests were applied when data were not normally distributed (Kolmogorov–Smirnov test for normality). Changes and differences were considered statistically significant at *p* < 0.05.

No formal missing data imputation was required. Annual average pollutant concentrations were obtained from official regional monitoring reports, which provide complete datasets for each year. Clinical data were based on medical records coded with ICD‐10 at the time of presentation, and all eligible cases were included.

No sampling strategy was used, as all eligible emergency department visits during the study period were included. Therefore, no weighting or adjustments for sampling design were necessary in the analyses.

Sensitivity analyses were conducted by excluding noninflammatory cases and conjunctivitis, the most frequent and clinically less severe subgroup. The associations between pollutant concentrations and AOICs prevalence persisted and were strengthened in these restricted models, indicating the robustness of the findings.

## 3. Results

A total of 94,836 patients attended the emergency department between 01/01/2011 and 31/12/2019. Of this total, 60,586 (63.9%) presented with AOICs. The recorded cases included 8533 of blepharitis (14.1%), 436 of orbital inflammation (0.7%), 611 of lacrimal system inflammation (1.0%), 28,326 of conjunctivitis (46.8%), 16,002 of keratitis (26.4%), 3419 of uveitis with retinitis (5.6%), 198 of endophthalmitis (0.3%), 2594 of optic neuritis (4.3%), and 467 of scleritis (0.8%). As this was a retrospective study based on routinely collected medical records, there was no active patient recruitment and thus no nonparticipation. No individual‐level demographic or social characteristics were available, as the analysis was based exclusively on aggregated annual diagnostic data extracted from hospital records. All exposures (pollutant concentrations and temperature) and the outcome (the annual AOICs prevalence) were analyzed as continuous variables. No categorization was applied, and therefore no category boundaries are reported. Table 3 shows the significant associations between mean annual concentrations of pollutants and/or temperature and the prevalence of AOICs. The cells marked plus (i.e., a direct positive correlation) or minus (i.e., a negative correlation) identify the pollutants that were included in the regression analysis. The prevalence (*y*) of all AOICs among all the cases that attended the emergency department was significantly associated with the mean annual concentration of formaldehyde. This association is best described by Equation (1) in Table 4. Conjunctivitis was the cause of inflammation in over 46% of all AOICs. The high volume of conjunctivitis cases could obscure the detection of significant associations involving less common AOICs. Therefore, the analysis was repeated after excluding noninflammatory conditions and conjunctivitis.

**TABLE 3:** The significant (*p* < 0.05) correlation coefficients [*r*] between mean annual atmospheric concentration of pollutants, atmospheric temperature, and the prevalence of specific acute inflammatory conditions among all cases attending with an ophthalmic inflammatory condition over the period 2011–2019.

	All	Bleph	LSI	Orbital	Con	Scl	Ker	U and R	Endo	ON
SO_2_		—	—		+	+●		+	+	—
NO_2_			—							—
NOx			—	—						—
CO		—	—	—	+	+●	—+●	+●	+●	—
PM_10_			—	—	+			+	+	—
PM_2.5_			—	—				+●	+	—
Pb(PM_10_)						—		+		
As(PM_10_)	—●									
BaP(PM_10_)										—●
PAHs(PM_10_)									+●	—
Formaldehyde	+		—						+	—
Temperature						—●		—●	—	

*Note:* + = Significant positive correlation between mean annual atmospheric concentration of pollutant or temperature and the prevalence of the acute ophthalmic inflammatory condition. — = Significant negative correlation between mean annual atmospheric concentration of pollutant or temperature and the prevalence of the acute ophthalmic inflammatory condition. ● Sign of significant correlation after excluding all cases of conjunctivitis. Bleph, blepharitis; Orbital, orbital inflammation; Con, conjunctivitis; Scl, scleritis; Ker, keratitis; U and R, uveitis with retinitis; Endo, endophthalmitis.

Abbreviations: LSI, lacrimal system inflammation; ON, optic neuritis.

**TABLE 4:** Statistically significant least squares expressions between mean annual prevalence of ophthalmic inflammatory condition (*y*), mean annual atmospheric concentrations of pollutants and temperature among cases attended between 2011 and 2019, mean (±sd, 95% CI limits), and limits of agreement between the estimated and actual prevalence values.

Condition	Expressions and associated correlation coefficients	Actual mean (±sd, 95% CI limits)	Estimated mean (±sd, 95% CI limits)	LoA
*Section 1: As a percentage of all cases (inflammatory and noninflammatory)*				
I	*y* = 1.999*x*_1_ + 58.021, *r* ^2^= 0.629 Equation (1)	64.05 (±2.46, 62.44–65.66)	64.05 (±2.46, 62.45–65.65)	±4.61
II	*y* = 76.66*x*_2_ − 0.765*x*_3_ + 0.732, *r* ^2^ = 0.711 Equation (A)	30.25 (±3.49, 27.97–32.53)	31.84 (±3.10, 29.21–33.87)	±3.69
II	*y* = 102.75*x*_2_ − 0.503*x*_3_ − 0.544*x*_4_ + 5.080, *r* ^2^ = 0.766. Equation (B)	30.25 (±3.49, 27.97–32.53)	30.25 (±3.04, 28.27–32.23)	±3.31
II	*y* = 55.61*x*_2_ + 0.970*x*_4_ − 8.862, *r* ^2^ = 0.703^∗^. Equation (2)	30.25 (±3.49, 27.97–32.53)	30.26 (±2.93, 28.35–32.17)	±3.71
III	*y* = 0.127*x*_3_+ 4.929*x*_5_ + 1.950, *r* ^2^ = 0.675^∗^. Equation (3)	3.64 (±0.38, 3.39–3.89)	3.63 (±0.36, 3.42–3.84)	±0.37
III	*y* = 0.059*x*_3_ + 2.330*x*_5_ − 0.249*x*_4_ + 5.377, *r* ^2^ = 0.739^∗^. Equation (4)	3.64 (±0.38, 3.39–3.89)	3.64 (±0.34, 3.42–3.86)	±0.29
IV	*y* = 0.037*x*_1_ + 0.003*x*_4_ + 0.073*x*_6_ − 0.044*x*_7_ + 0.010*x*_3_ − 0.399, *r* ^2^ = 0.744 Equation (C)	0.22 (±0.09, 0.16–0.28)	0.18 (±0.09, 0.12–0.24)	±0.08
IV	*y* = 0.037*x*_1_ − 0.069*x*_4_ + 0.026*x*_6_ − 0.035*x*_3_ + 0.437, *r* ^2^ = 0.814 Equation (D)	0.22 (±0.09, 0.16–0.28)	0.32 (±0.07, 0.27–0.37)	±0.23

*Section 2: As a percentage of all inflammatory cases only*				
II	*y*_1_ = 69.51*x*_2_ + 1.328*x*_4_ − 2.905, *r* ^2^ = 0.704^∗^ Equation (5)	47.15 (±4.30, 44.34–49.96)	47.17 (±3.62, 44.81–49.53)	±4.60
II	*y*_1_ = 78.467*x*_2_ − 0.594*x*_3_ + 13.027, *r* ^2^ = 0.682 Equation (^∗∗^)	47.15 (±4.30, 44.34–49.96)	47.16 (±3.53, 44.85–49.47)	±4.75
II	*y*_1_ = 2.535*x*_3_ + 3.970*x*_4_ − 23.763, *r* ^2^ = 0.659^∗^ Equation (6)	47.15 (±4.30, 44.34–49.96)	47.18 (±3.52, 44.88–49.48)	±4.80
III	*y*_1_ = 0.136*x*_3_ + 16.427*x*_7_ + 3.539, *r* ^2^ = 0.704^∗^ Equation (7)	5.68 (±0.52, 5.34–6.02)	5.67 (±0.44, 5.38–5.96)	±0.56
III	*y*_1_ = 0.03*x*_3_ + 12.381*x*_5_ − 0.388*x*_4_ + 8.874, *r* ^2^ = 0.804^∗^ Equation (8)	5.68 (±0.52, 5.34–6.02)	5.67 (±0.47, 5.36–5.98)	±0.44
III	*y*_1_ = 13.80*x*_5_ − 0.449*x*_4_ + 9.815, *r* ^2^ = 0.799^∗^ Equation (9)	5.68 (±0.52, 5.34–6.02)	5.67 (±0.47, 5.36–5.98)	±0.44
III	*y* = 0.054*x*_3_ − 0.427*x*_4_ + 9.346, *r* ^2^ = 0.773^∗^ Equation (10)	5.68 (±0.52, 5.34–6.02)	5.67 (±0.46, 5.37–5.97)	±0.48
IV	*y*_1_ = 0.059*x*_1_ − 0.087*x*_4_ + 1.034, *r* ^2^ = 0.687^∗^ Equation (11)	0.34 (±0.14, 0.25–0.43)	0.31 (±0.12, 0.24–0.38)	±0.21
IV	*y*_1_ = 0.027*x*_6_ − 0.059*x*_4_ + 0.139, *r* ^2^ = 0.766^∗^ Equation (12)	0.34 (±0.14, 0.25–0.43)	0.35 (±0.12, 0.27–0.43)	±0.13
IV	*y*_1_ = 0.012*x*_7_ − 0.087*x*_4_ + 0.700, *r* ^2^ = 0.675^∗^ Equation (13)	0.34 (±0.14, 0.25–0.43)	0.32 (±0.12, 0.24–0.40)	±0.25
IV	*y*_1_ = 0.045*x*_1_ + 0.042*x*_6_ − 0.059*x*_3_ − 0.121*x*_4_ + 0.872 *r* ^2^ = 0.914 Equation (D)	0.34 (±0.14, 0.25–0.43)	0.31 (±0.13, 0.43–0.39)	±0.24

*Section 3: As a percentage of all inflammatory cases excluding conjunctivitis*				
III	*y*_2_ = 0.823*x*_3_ + 4.133*x*_2_ − 0.146*x*_7_ − 0.270*x*_4_ + 7.044, *r* ^2^ = 0.943	10.84 (±1.53, 9.84–11.84)	10.32 (±1.73, 9.19–11.45)	±1.01
III	*y*_2_ = 0.688*x*_3_ − 2.07*x*_2_ + 3.588, *r* ^2^ = 0.910	10.84 (±1.53, 9.84–11.84)	10.83 (±1.46, 9.87–11.79)	±0.92
III	*y*_2_ = 0.772*x*_3_ + 6.455*x*_2_ − 0.162*x*_7_ + 4.880, *r* ^2^ = 0.936 Equation (^∗∗∗^)	10.84 (±1.53, 9.84–11.84)	10.82 (±1.49, 9.85–11.79)	±0.78
IV	*y* = 0.151*x*_1_ + 0.028*x*_7_ − 1.021, *r* ^2^ = 0.891^∗^ Equation (14)	0.64 (±0.29, 0.45–0.83)	0.56 (±0.28, 0.36–0.76)	±0.50
IV	*y*_2_ = 0.126*x*_1_ + 0.029*x*_7_ + 0.082*x*_4_ − 1.643, *r* ^2^ = 0.847^∗^ Equation (15)	0.64 (±0.29, 0.45–0.83	0.75 (±0.24, 0.58–0.92)	±0.53
V	*y*_2_ = 13.976 − 0.446*x*_1_ − 0.115*x*_7_, *r* ^2^ = 0.843^∗^ Equation (16)	7.95 (±1.05, 7.26–8.64)	7.95 (±0.96, 7.32–8.58)	±0.75
VI	*y*_2_ = 5.676 − 0.187*x*_1_ − 0.070*x*_8_, *r* ^2^ = 0.814^∗^ Equation (17)	1.83 (±0.47, 1.52–2.14)	1.92 (±0.48, 1.61–2.23)	±0.62
VI	*y*_2_ = 8.089 − 0.168*x*_1_ − 0.073*x*_8_ − 10.990*x*_2_ + 0.291*x*_3_, *r* ^2^ = 0.988 Equation (E)	1.83 (±0.47, 1.52–2.14)	1.97 (±0.61, 1.57–2.37)	±0.80
VII	*y*_2_ = 35.826*x*_2_ + 31.162, *r* ^2^ = 0.495. Equation (18)	50.03 (±3.09, 48.01–52.04)	50.03 (±2.18, 48.61–51.45)	±4.30

*Note:* LoA = limits of agreement between estimated and actual prevalence values (±1.96SD), *x*_1_ = mean annual atmospheric concentration of formaldehyde, *x*_2_ = mean annual atmospheric concentration of CO, *x*_3_ = mean annual atmospheric concentration of SO2, *x*_4_ = mean annual temperature, *x*_5_ = mean annual atmospheric concentration of Pb(PM10), *x*_6_ = mean annual atmospheric concentration of PM2.5, *x*_7_ = mean annual atmospheric concentration of PM10, *x*_8_ = mean annual atmospheric concentration of NOx. I = all inflammatory conditions. II = conjunctivitis, III = uveitis with retinitis, IV = endophthalmitis, V = optic neuritis, VI = lacrimal system inflammations, VII = keratitis, *y* = [Total number of all inflammatory cases/Total number of all cases that attended the emergency department] × 100%, *y*_1_ = [Total number of cases/total number of all inflammatory cases] × 100%, *y*_2_ = [Total number of cases/total number of all inflammatory cases excluding conjunctivitis] × 100%, *n* = 9, *p* < 0.05.

^∗^Expressions where all variance inflation factors (VIF) were below 4. The VIF values are Equation (2), 1.77 (for both variables); Equation (3), 1.77 (for both variables); Equation (4), 3.69 (*x*_4_), 3.92 (*x*_3_), 1.83 (*x*_5_); Equation (5), 1.74 (for both variables); Equation (6), 3.56 (for both variables); Equation (7), 1.77 (for both variables); Equation (8), 3.92 (*x*_3_), 3.69 (*x*_4_), 1.83 (*x*_5_); Equation (9), 1.66 (for both variables); Equation (10), 2.56 (for both variables); Equation (11), 1.70 (for both variables); Equation (12), 2.00 (for both variables); Equation (13), 1.79 (for both variables); Equation (14), 1.92 (for both variables); Equation (15), 2.30 (*x*_1_), 1.93 (*x*_6_), 1.34 (*x*_4_); Equation (16), 1.92 (for both variables); Equation (17), 2.20 (for both variables).

Multiple linear regression analyses revealed that the prevalence of conjunctivitis, uveitis with retinitis, endophthalmitis, optic neuritis, lacrimal system inflammation, and keratitis was significantly associated with mean annual concentrations of various pollutants and/or temperature. The expressions describing these significant associations are shown in Table 4.

The actual and estimated prevalence of the AOICs, according to the best‐fitting expressions, are presented in Table 4. The mean difference between the actual and estimated values, and the LoA (LoA = 1.96 ×SD) between these values are also noted in Table 4. The variance inflation factors (VIFs) of some variables in the tabled expressions are above 15. These results can be interpreted as having high levels of multicollinearity where the concentration of pollutants and/or temperature should not be considered as independent variables [21–23]. The likelihood of a multicollinearity problem is low when the VIF values are below 4, and these expressions are marked with a caret (^∗^) in Table 4. The corresponding VIF values are included in the notes accompanying Table 4.

Multiple linear regression analyses did not reveal any significant association between the prevalence of scleritis, orbital inflammation, or blepharitis and mean pollutant levels and/or temperature.

## 4. Discussion

Previous studies on pollution and ophthalmological conditions tended to focus on DED, conjunctivitis, and ocular surface disorders [14, 15, 17]. In contrast, the present study evaluated a broad spectrum of AOICs and identified significant associations with atmospheric pollutants, including formaldehyde, SO_2_, CO, PM_10_, and PM_2.5_. These findings are generally consistent with earlier reports linking air pollution to ocular inflammation, while extending previous observations to less frequently studied conditions such as endophthalmitis, optic neuritis, and lacrimal inflammation. Bourcier et al. analyzed emergency ophthalmology visits in relation to air pollution and weather conditions using regression‐based time‐series methods [24]. Khalaila et al. investigated the association between ambient temperature, PM, and conjunctivitis‐related emergency department visits using a case‐crossover design and conditional logistic regression models [25]. Wang et al. evaluated the short‐term effects of air pollutants on emergency room visits for eye diseases using generalized additive models (GAMs) with a quasi‐Poisson regression link and lagged exposure analyses [26].

However, these studies primarily focused on estimating associations, relative risks, or odds ratios rather than developing predictive equations. In contrast, the present study developed multivariable regression equations incorporating temperature and multiple pollutants, enabling direct estimation of the expected frequency of AOICs under different environmental conditions.

Previous studies have attempted to develop predictive models linking environmental factors and ocular diseases. Youn et al. developed a regression‐based prediction model for DED incidence using air pollutants (PM_10_, NO_2_, SO_2_, and CO) and meteorological variables, including temperature and humidity [27]. Similarly, Seo et al. proposed a conjunctivitis outpatient rate prediction model incorporating environmental and meteorological factors [28]. Unlike these studies, which focused on single ocular disorders, our models encompass a broad spectrum of AOICs.

**FIGURE 1:**
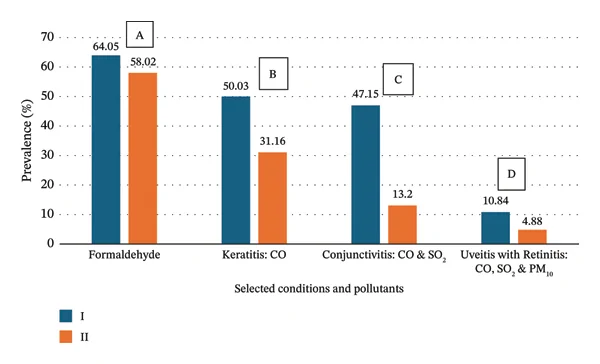
Estimated changes in the prevalence of selected conditions on condition levels of selected pollutants are nullified. Notes: I, prevalence over the period 2011–19. II, estimated prevalence after removing all traces of the noted pollutant(s). (A) Prevalence of all ocular inflammatory cases in population attending emergency eye department (Table 4, Equation (1)). (B) Prevalence of keratitis among the ocular inflammatory cases after excluding all cases of conjunctivitis (Table 4, Equation (18)). (C) Prevalence of conjunctivitis among all ocular inflammatory cases (Table 4, Equation (^∗∗^)). (D) Prevalence of uveitis with retinitis among the ocular inflammatory cases after excluding all cases of conjunctivitis (Table 4, Equation (^∗∗∗^)).

Among the pollutants evaluated, formaldehyde emerged as the most consistent predictor of the overall prevalence of AOICs. Therefore, its potential role deserves particular attention. The concentration of formaldehyde is the single atmospheric factor associated with the prevalence of all AOICs. According to Equation (1) in Table 4 (Section 1) and Figure 1, reducing the concentration of formaldehyde to zero should reduce the prevalence of all ophthalmic inflammatory conditions from 64% to 58%. The LoA of Equation (1), ±4.61%, can be interpreted as indicative of the confidence that can be placed on the estimated 6% reduction. Table 3 shows the prevalence of some AOICs correlated positively, while others correlated negatively, with atmospheric pollutant levels. This diversity of correlations will impact on the confidence of Equation (1). Tables 3 and 4 show that, besides temperature, the chief pollutants associated with the prevalence of the various ophthalmic inflammatory conditions are SO_2_, CO, PM_10_, and PM_2.5_.

To our knowledge, few studies have specifically examined the relationship between formaldehyde exposure and AOICs. Previous research has primarily focused on ocular irritation [29], ocular surface inflammation [30], and corneal injury [31]. In contrast, evidence regarding the role of formaldehyde in ophthalmic inflammatory conditions remains limited. Formaldehyde is known to induce oxidative stress and inflammatory responses in exposed tissues, which may provide a biological explanation for the consistent associations observed in the present study [29]. Therefore, the consistent association between formaldehyde and the prevalence of AOICs observed in the present study should be considered hypothesis‐generating and warrants further investigation.

Atmospheric temperatures are affected by pollutants that are naturally occurring and those released by industrial activity. Atmospheric temperature and pollutant concentrations are interrelated and therefore should not be considered mutually independent factors. Thus, the outcomes of multiple linear regression analyses, noted in Table 4, are expected to demonstrate relatively high VIF values. Nevertheless, a model with high VIF may still provide useful predictions when the associated correlation coefficient is high, or its predictions closely match actual results. The associations identified for individual AOICs are discussed below, beginning with conjunctivitis, the most common inflammatory condition in our cohort.

### 4.1. Conjunctivitis

The observed positive association between conjunctivitis and CO concentration may reflect the irritative and proinflammatory effects of air pollutants on the ocular surface [32]. Although CO is not a strong irritant at typical environmental levels, it often appears alongside other pollutants from traffic or industrial sources and may serve as an indicator of overall air pollution exposure [33]. Similarly, the positive correlation with temperature may contribute to the seasonal patterns, with warmer months facilitating greater pollutant dispersion or increased exposure due to behavioral factors such as outdoor activity [34]. These findings are consistent with previous studies linking air quality and weather conditions to fluctuations in conjunctival inflammation, particularly in urban environments [25, 35, 36]. Figure 1 shows that reducing the ambient levels of CO and SO_2_ has the potential to reduce the prevalence of conjunctivitis in the population attending the emergency eye hospital.

For example, Equation (2), A and B in Table 4 suggest that reducing atmospheric levels of these pollutants to zero could lower the prevalence of conjunctivitis. According to these models, the prevalence among all emergency cases would decrease from 30.25% (±3.49) to less than 1% at a mean annual temperature of 10°C. However, according to Equations (5) and (6), in Section 2 of Table 4, reducing atmospheric levels of CO and SO_2_ could lower the prevalence of conjunctivitis among all AOICs from an average of 47.15% (±4.30) to between 10.4% and 15.9% for a mean annual temperature of 10°C. Although the estimates vary, they show the same trend. Any strategy adopted to reduce the prevalence of conjunctivitis among all ophthalmic inflammatory cases will, by definition, be met with a concurrent rise in the prevalence of other conditions. Beyond conjunctivitis, significant associations were also identified for deeper inflammatory ocular disorders, particularly uveitis with retinitis.

### 4.2. Uveitis With Retinitis

Figure 1 shows that reducing exposure to CO, SO_2_, and PM_10_ should lower the prevalence of uveitis with retinitis. Exposure to SO_2_ and PM_10_ was significantly associated with the prevalence of uveitis with retinitis, whereas higher temperatures were linked to lower prevalence values. The association with PM supports the results from a study based in China [37]. However, the association with uveitis was stronger for PM_2.5_ as opposed to PM_10_ levels. The particularly strong influence of lead (Pb(PM_10_)) may suggest a toxic‐inflammatory mechanism involving oxidative stress or direct disruption of the blood–ocular barrier [38]. Similar associations between heavy metal exposure and retinal inflammation have been reported in environmental and toxicological studies [4, 34, 35, 39]. On a closer examination, the corresponding models in Section 2 of Table 4 generate conflicting estimates. According to Equations (3) and (7), reducing the atmospheric levels of the noted pollutants to zero should reduce the overall prevalence of uveitis with retinitis about 2%. However, according to Equations (4) and (8), reducing the levels of these pollutants to zero would have little impact on the prevalence when the mean annual temperature is approximately 10°C. The prevalence values are expected to rise when there is a concurrent fall in ambient temperature levels. This aligns with the findings that anterior uveitis cases rise during cooler winter months [40]. Unlike uveitis, endophthalmitis has rarely been investigated in relation to environmental pollution.

### 4.3. Endophthalmitis

Endophthalmitis is generally considered as a possible complication of intraocular surgery. To our knowledge, published studies investigating the effects of air pollution have primarily focused on ocular surface disorders, conjunctivitis, DED, allergic eye disease, and uveitis [4, 7, 8, 37, 41]. Evidence regarding endophthalmitis remains scarce. Nevertheless, according to Equation (C), the prevalence of endophthalmitis can be estimated within ±0.08% using the mean temperature and the concentrations of formaldehyde, SO_2_, PM_10_, and PM_2.5_. Equations (11)–(15) indicate that reducing the levels of formaldehyde, PM_10_, and PM_2.5_ should reduce the prevalence of endophthalmitis among inflammatory cases. According to Equation (D), there is a tendency toward a negative association with temperature and SO_2_ levels. Higher temperatures and SO_2_ levels were associated with lower estimated prevalence. Although SO_2_ is generally considered a harmful pollutant, its negative correlation with endophthalmitis rates may result from complex interactions with other environmental factors or an ecological bias [42]. Higher temperatures may reduce the viability of certain pathogens or influence human behavioral patterns, such as increased ventilation, thereby decreasing the risk of infections [43, 44]. This must be viewed with caution because according to Equation (C), the association with temperature and SO_2_ levels is positive. Turning to Equations (14) and (15), an association with SO_2_ levels was observed, and a positive association with temperature was observed, after excluding all cases of conjunctivitis. The high number of conjunctivitis cases (*n* = 28,326) appears to overshadow any links between endophthalmitis (*n* = 198) and environmental factors. Beyond these conditions, significant associations were also identified for several less common inflammatory ocular disorders.

### 4.4. Other Conditions

Further stratification of the dataset by the exclusion of both conjunctivitis and noninflammatory trauma cases revealed statistically significant regression models for optic neuritis, lacrimal inflammation, and keratitis. The models in Section 3 of Table 4 demonstrate high correlations (*r* ^2^ up to 0.988) with selected air pollutants, including formaldehyde, CO, SO_2_, and PM_10_. The prominence of keratitis in this group is highlighted in Figure 1, and according to Equation (18), nullifying the concentration of CO has the potential to reduce this prevalence to under 32%. The observed association between air pollutants and optic neuritis is consistent with the hypothesis that airborne toxins may contribute to optic nerve inflammation through immune‐mediated or neurotoxic mechanisms [45]. Equation (16) suggests that environmental exposures to formaldehyde and PM_10_ may disproportionately influence the prevalence of optic neuritis by targeting anatomically deeper ocular tissues. The stepwise narrowing of the case group enhanced the visibility of pollutant–disease associations. These findings underscore the importance of focusing on well‐defined inflammatory entities when investigating environmental influences on ocular morbidity, particularly those involving intraocular or neuro‐ophthalmic structures. Turning to Equation (17) and E, these models for the prevalence of lacrimal inflammation also include NO_x_. To date, there is limited published information directly linking air pollution with lacrimal system inflammation. While the impact of air pollution on the ocular surface, tear film stability, and dry eye symptoms is documented [7, 41], deeper periocular structures such as the lacrimal gland and nasolacrimal duct have received little attention in this context. The associations observed in this study suggest that these less frequently studied inflammatory conditions may be strongly influenced by environmental factors than previously recognized. However, Table 3 shows that the prevalence of optic neuritis and lacrimal inflammation is mainly negatively correlated with the concentrations of the selected air pollutants, and Equations (16), (17) and E predict increased prevalence of these conditions when pollutant levels approach zero. This implies that higher levels of the specified pollutants could offer some protection against these inflammatory conditions. On a purely speculative basis, lowering these pollutant levels may trigger events culminating in either optic neuritis or lacrimal inflammation.

These findings suggest that specific environmental pollutants, including formaldehyde, CO, SO_2_, PM_2.5_, and PM_10_, are significantly associated with the prevalence of various inflammatory ocular conditions. The strength and consistency of these associations increased when the analysis was limited to less common inflammatory conditions after excluding noninflammatory conditions and conjunctivitis. These results highlight the importance of environmental monitoring and its potential role in predicting or mitigating the risk of serious ocular inflammation. Further research is warranted to confirm these associations in larger cohorts and to explore underlying pathophysiological mechanisms.

## 5. Clinical Implications

This study indicates that certain air pollutants, particularly SO_2_, CO, PM_10_, and PM_2.5_, may significantly contribute to the development of inflammatory ocular diseases such as uveitis with retinitis, endophthalmitis, optic neuritis, and lacrimal inflammation. The improved strength and clarity of regression models after excluding noninflammatory and nonspecific conditions, such as conjunctivitis, suggest that these environmental factors have a more pronounced effect on deeper, more severe ocular inflammation. Clinicians should consider environmental exposure as a relevant risk factor, especially in susceptible individuals or in areas with high levels of air pollution. Preventive strategies, such as patient education and closer monitoring during periods of poor air quality, may help reduce the burden of environmentally driven ocular inflammation.

### 5.1. Study Limitations

This retrospective, single‐center study is subject to certain limitations. Diagnostic classification was based on existing medical records, which may vary in completeness and accuracy. Yearly aggregation of cases limited temporal resolution and precluded analysis of short‐term environmental effects. Additionally, exposure to air pollutants was estimated from regional monitoring stations and not linked to individual patient locations. Finally, as an ecological study, the analysis did not account for individual‐level confounders such as age, comorbidities, or socioeconomic factors. Therefore, the observed associations should not be interpreted as evidence of causal relationships between environmental factors and AOICs prevalence.

## 6. Future Directions

Future studies should explore the observed associations in larger, multicenter cohorts and include individual‐level clinical data to better account for potential confounders. More precise exposure assessment, based on residential data or personal monitoring, may improve model accuracy. Prospective research is also needed to clarify causal links and to better understand the biological mechanisms through which air pollutants contribute to inflammatory eye diseases.

## 7. Conclusion

Formaldehyde, CO, SO_2_, PM_2_._5_, and PM_10_ were significantly linked to inflammatory ocular conditions, with stronger associations after excluding noninflammatory cases and conjunctivitis. These findings highlight the importance of environmental monitoring in predicting or reducing serious eye inflammation, supporting further large‐scale research.

## Author Contributions

Conceptualization: Monika Sarnat‐Kucharczyk and Sudi Patel; data curation: Monika Sarnat‐Kucharczyk and Sudi Patel; formal analysis: Monika Sarnat‐Kucharczyk and Sudi Patel; investigation: Monika Sarnat‐Kucharczyk, Sudi Patel, and Dorota Wyględowska‐Promieńska; methodology: Sudi Patel; project administration: Monika Sarnat‐Kucharczyk and Sudi Patel; resources: Monika Sarnat‐Kucharczyk; software: Monika Sarnat‐Kucharczyk and Sudi Patel; supervision: Monika Sarnat‐Kucharczyk, Sudi Patel, and Dorota Wyględowska‐Promieńska; validation: Monika Sarnat‐Kucharczyk, Sudi Patel, and Dorota Wyględowska‐Promieńska; visualization: Monika Sarnat‐Kucharczyk, Sudi Patel, and Dorota Wyględowska‐Promieńska; writing–original draft: Monika Sarnat‐Kucharczyk and Sudi Patel; writing–review and editing: Monika Sarnat‐Kucharczyk, Sudi Patel, and Dorota Wyględowska‐Promieńska.

## Funding

This research did not receive any specific grant from funding agencies in the public, commercial, or not‐for‐profit sectors.

## Disclosure

The authors have nothing to report.

## Ethics Statement

No animal studies are presented in this manuscript. In accordance with the Bioethics Committee of the Medical University of Silesia, retrospective data analysis is not classified as a medical experiment and, therefore, does not require the approval of the Bioethics Committee. The studies were conducted in accordance with the local legislation and institutional requirements. Written informed consent for participation was not required from the participants or the participants’ legal guardians/next of kin in accordance with the national legislation and institutional requirements. No potentially identifiable images or data are presented in this study.

## Conflicts of Interest

The authors declare no conflicts of interest.

## Supporting Information

Additional supporting information can be found online in the Supporting Information section.

## Supporting information


**Supporting Information** STROBE Statement—Checklist of items that should be included in reports of cross‐sectional studies.

## Data Availability

The original contributions presented in the study are included within the article; further inquiries can be directed to the corresponding author.
